# Epiglottic Ulceration as an Initial Manifestation of Crohn's Disease

**DOI:** 10.1002/jgf2.70089

**Published:** 2025-12-12

**Authors:** Niina Yamashita, Yoshiki Morihisa, Hiroaki Nishioka

**Affiliations:** ^1^ Department of General Internal Medicine Kobe City Medical Center General Hospital Kobe Hyogo Japan; ^2^ Department of Gastroenterology Kobe City Medical Center General Hospital Kobe Hyogo Japan

**Keywords:** bamboo joint‐like appearance, Crohn's disease, epiglottic ulcer, mesalazine, prednisolone

## Abstract

Crohn's disease can present with various extraintestinal symptoms; however, laryngeal involvement, particularly epiglottic ulcers, is rare. A 52‐year‐old man presented with a sore throat. Laryngeal endoscopy revealed an epiglottic ulcer. He had no gastrointestinal symptoms. Further evaluation revealed a bamboo joint‐like appearance in the stomach and aphthous ulcers in the terminal ileum; thus, Crohn's disease was diagnosed. Treatment with prednisolone and mesalazine resulted in the resolution of symptoms without relapse. This case highlights that Crohn's disease can cause isolated epiglottic ulcers even in the absence of gastrointestinal symptoms and should be considered in the differential diagnosis of refractory throat ulcers.

## 1 Background

A sore throat is a common symptom that can result from various causes, including lesions of the epiglottis. The causes of epiglottic ulceration include inflammatory bowel diseases, autoimmune diseases, infections, neoplastic diseases, and trauma. Crohn's disease is an idiopathic inflammatory bowel disease characterized by transmural inflammation that can affect any part of the gastrointestinal tract, ranging from the oral cavity to the perianal area. It primarily affects the small and large intestines, leading to specific lesions such as longitudinal ulcers and a cobblestone appearance. Common clinical symptoms include abdominal pain, diarrhea, and weight loss. In addition to intestinal involvement, Crohn's disease can manifest as extraintestinal conditions, such as arthritis, ankylosing spondylitis, oral aphthous ulcers, erythema nodosum, pyoderma gangrenosum, iritis, uveitis, and primary sclerosing cholangitis [1]. However, epiglottic ulcers are rare.

In this report, we present a case of Crohn's disease accompanied by an intractable epiglottic ulcer without any obvious gastrointestinal symptoms.

## 2 Case Presentation

**FIGURE 1:**
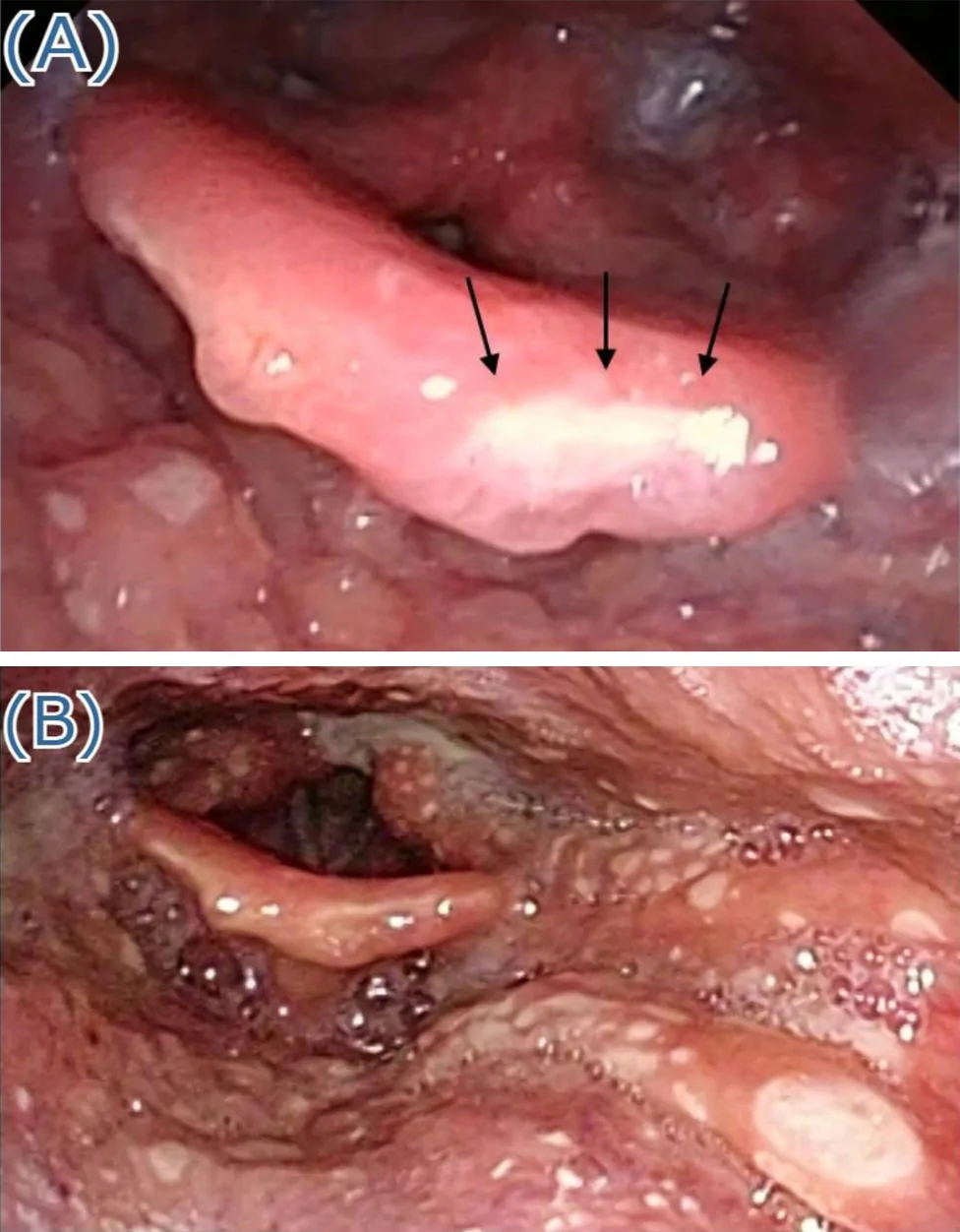
(A) Ulceration of the epiglottis observed at the first visit (black arrows). (B) Multiple ulcers in the hypopharynx and larynx observed 2 weeks after the first visit.

A previously healthy 52‐year‐old man presented to our hospital with a 1‐month history of sore throat that gradually worsened. The patient reported no fever, abdominal pain, diarrhea, hematochezia, or joint pain. The patient's vital signs were normal. Physical examination revealed no aphthous ulcers in the oral cavity. The tonsils were neither red nor swollen. No neck lymphadenopathy was noted. The abdomen was soft and flat, without tenderness, and no swelling or erythema was noted in the joints. No rashes were observed in the genitals. Laboratory tests showed the following results: white blood cell count, 5200/μL; hemoglobin, 13.9 g/dL; platelet count, 234,000/μL; C‐reactive protein, 0.03 mg/dL; and erythrocyte sedimentation rate, 37 mm/h. Autoantibody tests for myeloperoxidase‐antineutrophil cytoplasmic antibody and proteinase 3‐antineutrophil cytoplasmic antibody, anti‐desmoglein 1, anti‐desmoglein 3, and anti‐BP180 yielded negative results. The infection workup was negative for syphilis, hepatitis B, and human immunodeficiency virus. Epstein–Barr virus and cytomegalovirus serologies indicated past infections. Laryngeal endoscopy performed at the initial visit confirmed the presence of an ulcerative lesion with irregular margins in the epiglottis (Figure 1A). Histopathological examination of the epiglottic biopsy specimen revealed infiltration of inflammatory cells, specifically neutrophils and lymphocytes, within and beneath the epithelium. There was no evidence of noncaseating granulomas or malignant cells.

**FIGURE 2:**
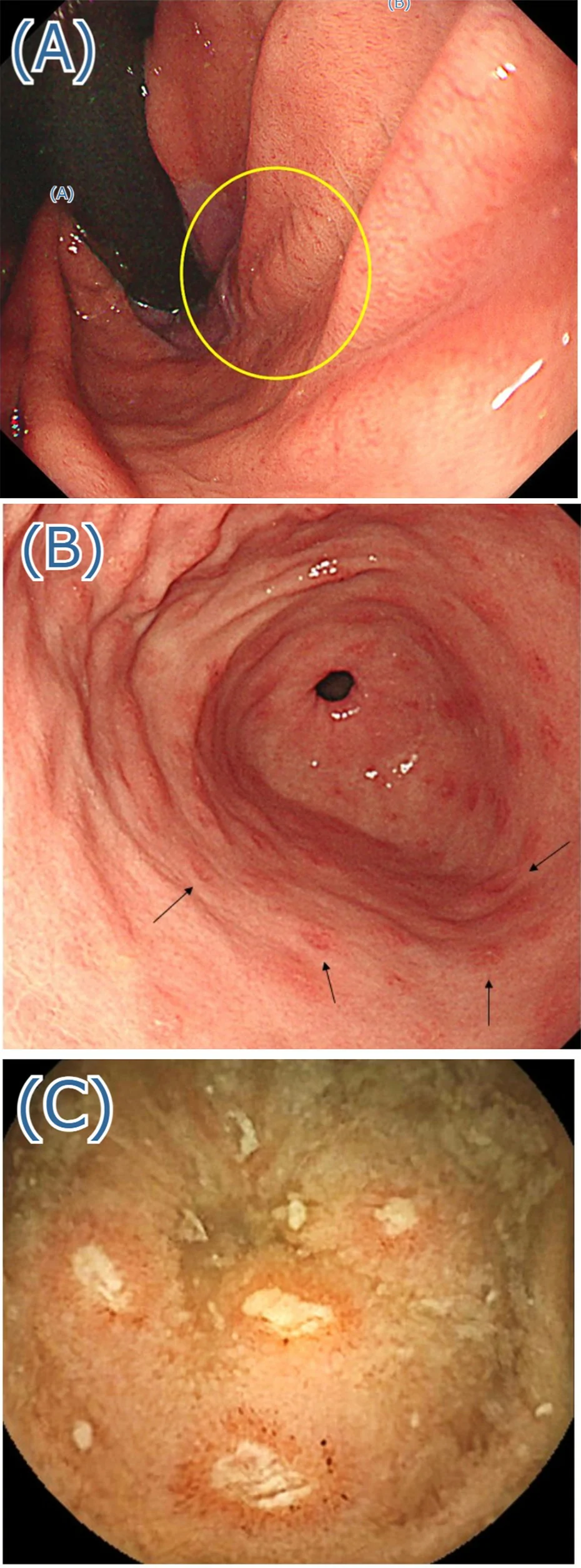
(A) bamboo‐joint appearance in the stomach observed using upper gastrointestinal endoscopy (yellow circle). (B) Multiple aphthous ulcers in the gastric antrum observed using upper gastrointestinal endoscopy (black arrows). (C) Multiple aphthous ulcers with irregular margins are present throughout the small intestine, particularly increasing in number toward the terminal ileum, as observed using capsule endoscopy.

Treatment with amoxicillin, valacyclovir, and miconazole was attempted but was ineffective. Two weeks later, multiple ulcerative lesions appeared in the hypopharynx and larynx (Figure 1B). By the fourth week, multiple aphthous ulcers had developed on the lips and tongue. Considering the possibility of inflammatory bowel disease or Behçet's disease, gastrointestinal examination was performed. Upper gastrointestinal endoscopy revealed a bamboo joint‐like appearance in the gastric body (Figure 2A) and aphthous ulcers in the gastric antrum (Figure 2B). Colonoscopy revealed small aphthous ulcers in the cecum, from which biopsy was performed. Histopathological examination revealed moderate infiltration of inflammatory cells, specifically lymphocytes and plasma cells, into the lamina propria; however, no granuloma formation was observed. Capsule endoscopy identified multiple aphthous ulcers with irregular margins throughout the small intestine (Figure 2C), which increased in number toward the terminal ileum.

Based on these findings, the patient was strongly suspected of Crohn's disease. Induction therapy for Crohn's disease was initiated with prednisolone (30 mg) and mesalazine (3000 mg), which led to rapid improvement in the patient's sore throat. Laryngeal endoscopy performed 2 weeks after treatment initiation revealed complete healing of the ulcers. The prednisolone dose was gradually tapered, and eventually, it was discontinued. Azathioprine (25 mg) was introduced as maintenance therapy, along with mesalazine (3000 mg). Capsule endoscopy performed 1 year later showed that the ulcers in the small intestine had resolved. The patient has remained in remission for more than 18 months. We diagnosed the patient with a strong suspicion of Crohn's disease accompanied by epiglottic ulcers.

## 3 Discussion

The patient's clinical course highlights two important observations. First, epiglottic ulcers may develop in patients with Crohn's disease. Second, laryngeal symptoms can precede gastrointestinal symptoms in patients with Crohn's disease.

Head and neck manifestations of Crohn's disease are relatively uncommon, with incidences ranging from 0.5% to 13% [2]. The oral cavity is the most frequently affected area in the head and neck. Oral aphthous ulcers, particularly those on the tongue, buccal mucosa, and palate, are the most common lesions observed. However, laryngeal involvement, particularly the presence of ulcers in the epiglottis, is rare. To date, only four cases of Crohn's disease accompanied by epiglottic ulcers have been documented [3, 4, 5, 6]. Other causes of epiglottic ulceration include autoimmune diseases, such as Behçet's disease, systemic lupus erythematosus, pemphigus vulgaris, and bullous pemphigoid; infections, such as herpes simplex virus, varicella‐zoster virus, human immunodeficiency virus, histoplasmosis, and tuberculosis; and neoplastic diseases, such as laryngeal carcinoma and T‐cell lymphoma. Trauma due to radiation or nasogastric intubation should also be considered.

Our patient did not meet the criteria for a definitive diagnosis of Crohn's disease according to the Japanese diagnostic criteria [7]. No non‐caseating granulomas were detected in the biopsy specimens, and typical findings such as longitudinal ulcers or a cobblestone appearance were absent. Therefore, this case is classified as “suspected Crohn's disease” rather than a “definitive” diagnosis. However, at the time of the initial diagnosis of Crohn's disease, some studies have reported that only 40% to 55% of patients show histological evidence of non‐caseating granulomas, which are specific to Crohn's disease [8]. In our case, these specific findings were not present, likely because the intestinal lesions may have been at a very early stage of the condition. The endoscopic evaluation revealed several findings that strongly suggested Crohn's disease. These included a bamboo joint‐like appearance of the gastric body, multiple aphthous ulcers in the gastric antrum, an increase in aphthous lesions toward the terminal ileum, and the presence of aphthous lesions in the cecum. The bamboo joint‐like appearance is recognized as a relatively specific marker for Crohn's disease affecting the stomach [8, 9]. Additionally, the distribution pattern of the aphthous lesions, which tends to increase in number in the terminal ileum, is highly characteristic of Crohn's disease and distinguishes it from other inflammatory or infectious conditions [10]. In this case, no malignancy was detected on biopsy. Treatment with antimicrobial, antiviral, or antifungal drugs did not yield any results. Collectively, the characteristic endoscopic results and the patient's positive response to treatment raise a strong suspicion that Crohn's disease is the most likely underlying etiology.

In Crohn's disease, extraintestinal symptoms can occur before gastrointestinal symptoms. For instance, oral manifestations, such as aphthous ulcers or pain in the mouth and gums, can occur in 26% to 60% of cases before gastrointestinal symptoms [11, 12]. These oral manifestations can develop at any stage of the disease and may be the first symptoms noticed by the patients [3]. Additionally, the severity of oral manifestations does not always correlate with the severity of gastrointestinal symptoms. In cases of Crohn's disease with laryngeal involvement, patients may exhibit symptoms affecting the larynx, such as a sore throat, pain during swallowing, changes in voice, chronic cough, and dysphagia. However, only a few instances have documented laryngeal symptoms as the initial signs of Crohn's disease, appearing before any obvious gastrointestinal complaints [5, 13]. In our case, the patient first presented with a sore throat but did not report abdominal pain, diarrhea, or hematochezia.

## 4 Conclusion

This case demonstrates that epiglottic ulcers cause a sore throat and can develop in patients with Crohn's disease, and that laryngeal symptoms may appear before gastrointestinal issues. When patients present with persistent laryngeal ulcers, physicians should consider Crohn's disease as a potential differential diagnosis and perform gastrointestinal evaluations, even in the absence of gastrointestinal symptoms.

## Author Contributions

All authors contributed to the conception and design of the study. N.Y. and H.N. prepared the main manuscript and conducted the literature review. Y.M. provided endoscopy examination data and assisted in drafting the manuscript. H.N. supervised the project. All authors reviewed and approved the final manuscript.

## Funding

This work was supported by the Matsumoto Allergy and Immunology Research Fund from Kobe City Medical Center General Hospital.

## Ethics Statement

Written informed consent was obtained from the patient for publication of this case report and accompanying images. According to our institutional policy, approval from the ethics committee is not required for this type of case report.

## Conflicts of Interest

The authors declare no conflicts of interest.

## Data Availability

Data sharing not applicable to this article as no datasets were generated or analysed during the current study.
